# Mapeamento com Software *Coherent* para Ablação de *Flutter* Atrial Atípico – Um Passo à Frente na Compreensão do Mecanismo da Arritmia

**DOI:** 10.36660/abc.20201311

**Published:** 2021-11-22

**Authors:** Pedro A. Sousa, Sérgio Barra, Mariana Pereira, Luís Elvas

**Affiliations:** 1 Centro Hospitalar e Universitário de Coimbra Cardiology Department Pacing & Electrophysiology Unit Coimbra Portugal Pacing & Electrophysiology Unit, Cardiology Department, Centro Hospitalar e Universitário de Coimbra, Coimbra – Portugal; 2 Hospital da Luz Arrábida V Cardiology Department Vila Nova de Gaia Portugal Hospital da Luz Arrábida V - Cardiology Department, Vila Nova de Gaia – Portugal; 3 Royal Papworth Hospital NHS Foundation Trust Cardiology Department Cambridge Reino Unido Royal Papworth Hospital NHS Foundation Trust - Cardiology Department, Cambridge – Reino Unido

**Keywords:** Flutter Atrial/etiologia, Condução, Arritmias Cardíacas, HD Coloring, Coherent Mapping, Técnicas Eletrofisiológicas Cardíaca/métodos

## Apresentação do caso

**Figura 1:**
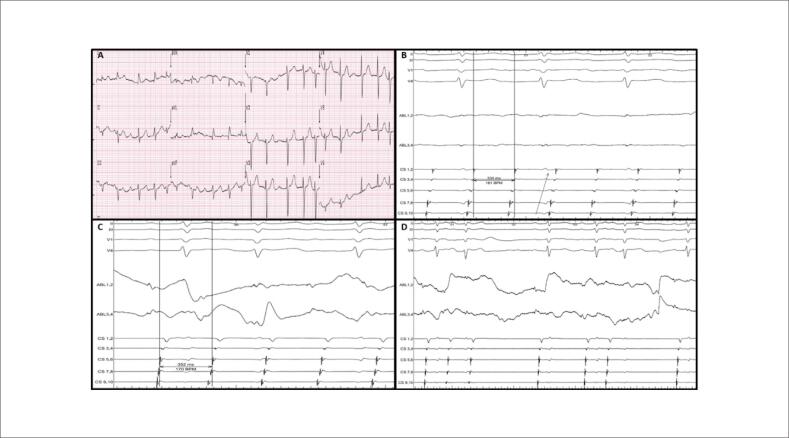
Eletrocardiograma de doze derivações (A) e traçados intracardíacos obtidos durante o FLA (B) e durante as aplicações de radiofrequência (C e D).

**Figura 2:**
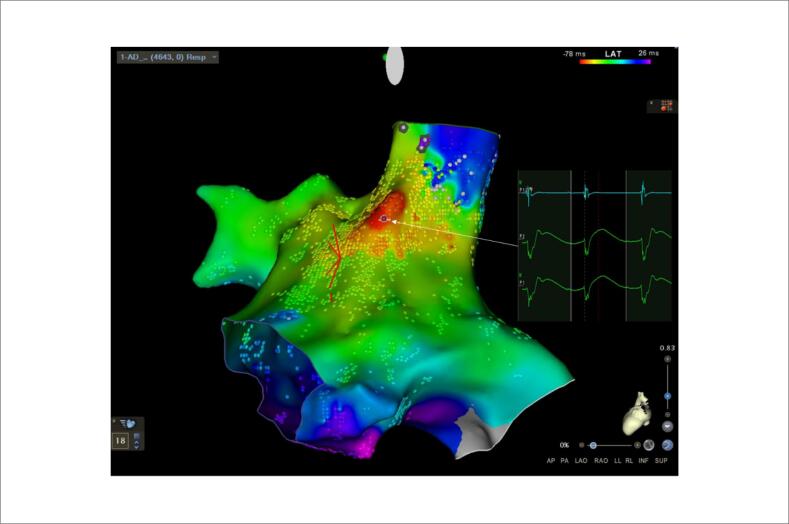
Mapa de ativação de alta densidade do AD adquirida durante FLA com uma TCL de 330ms. O mapa foi realizado com o software HD Coloring, e incluiu 4.643 pontos, com 32% da TCL e exibiu uma alta ativação septal centrífuga (vermelho indica as áreas com LAT mais precoce, enquanto laranja, amarelo, verde, azul e roxo indicam ativação progressivamente retardada) Os eletrogramas unipolares apresentaram uma deflexão inicial em “r”. FLA: flutter atrial; AD: átrio direito; TCL: duração do ciclo de taquicardia.

**Figura 3:**
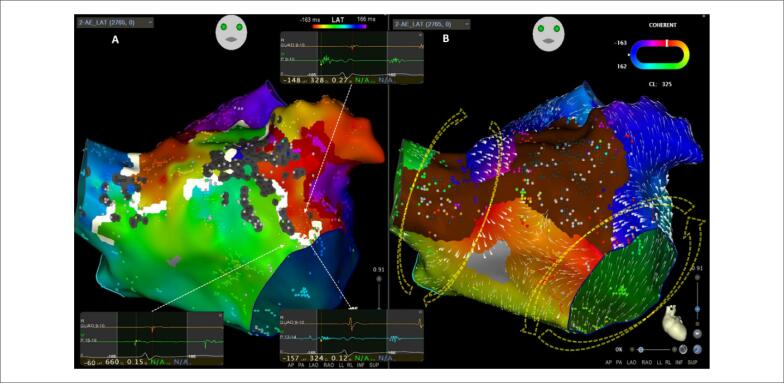
Mapas de ativação atrial esquerda realizados com o cateter PentaRay^®^, incluindo 2.765 pontos e 330ms da duração do ciclo da taquicardia. O Mapa A foi realizado com o software HD Coloring (EML e EEML definidos em 75% e 25%, respectivamente), com configurações de cicatriz bipolar em 0,03mV e um tamanho de área de cicatriz igual a 1 exibida como marcadores na cor cinza. O mapa de ativação revelou três áreas distintas de pontos LAT iniciais – no teto, perto da VM anterior e no AAE – e duas zonas de provável reentrada, como definido por uma diferença entre pontos adjacentes superior a 75% da TCL (o Limiar EML) – no teto e entre o AAE e a VM, respectivamente. Havia uma provável linha de bloqueio de condução (exibida como uma linha branca) da VM até quase a VPSD, uma vez que a diferença entre os pontos LAT adjacentes em ambos os lados da linha eram maiores que 25% do ciclo de taquicardia mapeado. O mapa B foi realizado com o algoritmo de mapeamento Coherent, revelando uma zona SNO (condução lenta ou sem condução) (exibida em marrom) da VPSD até a face anterior da válvula mitral e também em direção ao AAE. Com o vetor de velocidade fixado em 17 (a velocidade lenta é representada por vetores mais grossos), a condução foi observada através da parede anterior próxima à VPSD – um circuito no sentido horário (seta amarela tracejada) ao redor das veias pulmonares direitas. O Coherent também revelou uma pequena área de condução lenta (eletrogramas fracionados como mostrado no Mapa A) perto da válvula mitral, sugestiva de um segundo circuito – uma onda de propagação no sentido anti-horário em torno da VM (seta amarela tracejada). EML: early meets late; EEML: extended early meets late; LAT: Tempo de ativação local; VM: válvula mitral; AAE: apêndice atrial esquerdo; TCL: duração do ciclo de taquicardia; VPSD: veia pulmonar superior direita.

Uma mulher de 86 anos com antecedentes de fibrilação atrial paroxística foi encaminhada para ablação de *flutter* auricular (FLA) atípico. Ela nunca havia sido submetida a ablação por cateter antes. O ecocardiograma transtorácico revelou fração de ejeção do ventrículo esquerdo de 65%, átrio esquerdo discretamente dilatado (42 mm) e regurgitação mitral moderada. O ECG de 12 derivações mostrou ondas F positivas em V1 e nas derivações de membros inferiores, ondas F isoelétricas em DI e onda F negativa em aVL ( Figura 1A ). Dada a ativação concêntrica no seio coronário (SC), com uma duração do ciclo de taquicardia (TCL, *tachycardia cycle length* ) de 330ms, ( Figura 1B ) um mapa de ativação atrial direito (AD), realizado com o cateter PentaRay^®^, foi inicialmente analisado com a ferramenta *HD Coloring* (CARTO® 3V7, Biosense Webster, CA, EUA). Foi revelada uma ativação precoce no septo interatrial com apenas 1/3 da TCL ( Figura 2 ). Um mapa de ativação do átrio esquerdo (AE) subsequente revelou uma grande área com cicatriz e provável bloqueio de condução em quase toda a parede anterior representada como uma linha branca de acordo com o recurso da ferramenta *Extended Early Meets Late* (EEML), e várias áreas com tempo de ativação local (LAT, *local activation time* ) precoces – no apêndice atrial esquerdo (AAE), na face anterior da válvula mitral (VM) e no teto próximo ao segmento anterior da veia pulmonar superior direita (VPSD). Ele também mostrou duas zonas de reentrada, como estabelecido pelo recurso da ferramenta *Early Meets Late* (EML) ( Figura 3A e S-3 A ). Esses mapas sugeriam um provável circuito ao redor das veias pulmonares direitas, mas não explicavam a ativação da cor no AAE e na VM. Um novo algoritmo de mapeamento – *Coherent* (CARTO® 3V7, Biosense Webster) revelou o circuito ( Figura 3B e S-3 B ). Qual é o mecanismo desse FLA atípico?

## Discussão

Este caso destaca algumas características interessantes.

Primeiramente, tendo em consideração a ativação concêntrica no SC ( Figura 1B ), o mapeamento foi realizado inicialmente no AD com o *software HD Coloring* . O mapa bipolar revelou eletrogramas normais, definidos como voltagem acima de 0,3mV, na maior parte da câmara ( Figura S-2 A e S-2 B ). Um mapa de ativação do AD de alta densidade revelou os pontos de LAT mais precoces no septo interatrial ( Figura 2 ). A onda de propagação foi consistente com uma origem focal, mas como o sinal unipolar tinha uma deflexão inicial “r” e apenas 1/3 da TCL estava contido nessa câmara, um circuito exclusivamente no AD foi descartado ( Vídeo suplementar 1 ).

Um mapa bipolar do AE foi subsequentemente realizado, revelando uma disfunção no AE, com área de cicatriz densa e extensa na parede anterior, definida por uma voltagem abaixo de 0,1mV, e algumas áreas de cicatriz irregular na parede posterior. ( Figura S-2 A e S-2 B ) O mapa de ativação de alta densidade realizado com o software *HD Coloring* ( Figura 3A e S-3 A ), compreendendo toda a TCL, exibiu várias áreas de ativação precoce (no AAE, a face anterior da VM e o teto próximo ao segmento anterior da VPSD). Duas áreas de reentrada, como definido pelo recurso da ferramenta EML, também foram observadas – uma do AAE para a MV e outra no teto. Tanto a ferramenta EML quanto a EEML dependem do ciclo de taquicardia mapeado (que em nosso caso correspondeu à TCL), e são estabelecidos levando-se em consideração a diferença do LAT entre pontos adjacentes.^1 , 2^ Neste caso, uma vez que a diferença entre os pontos de LAT foi maior do que 25% da TCL (82ms = 0.25 * 330ms), uma linha branca foi mostrada da VM à VPSD (poupando apenas uma pequena parte na parede anterior, próximo à VPSD) e também na parede posterior próximo à VPSD, sugerindo prováveis linhas de bloqueio de condução.

O mapa de propagação do AE ( Vídeo Suplementar 2 ) sugeria um circuito ao redor das veias pulmonares direitas, com a onda de propagação movendo-se através da linha branca interrompida. No entanto, isso não explicava a ativação da cor no AAE e na VM. Mesmo depois de combinar os dois mapas ( Figura S-1 ), várias questões permaneceram sem explicação:

1. Como poderia haver uma onda de propagação na parede anterior, dada a presença de extensa área de cicatriz?
2. Como ocorreu a ativação simultânea de diferentes áreas do AE?
3. As duas áreas de reentrada – no teto posterior e do AAE para a VM - correspondem a dois circuitos independentes ou somente a um circuito com condução passiva na outra área?
4. No caso de circuitos independentes, onde estão eles localizados e como pode a propagação ocorrer pela face anterior da VM se, de acordo com a ferramenta EEML, esta aparece bloqueada (como demonstrado pela linha branca ininterrupta)?

Como relatado anteriormente,^2^ no recurso da ferramenta *HD Coloring* , cada LAT requer uma voltagem de apenas ≥ 0,03mV para ser integrada ao mapa de ativação. Esta é a razão pela qual foi possível ver uma onda de ativação na parede anterior, apesar da presença de cicatriz extensa (voltagem do eletrograma bipolar abaixo de 0,1mV). O *entrainment* poderia, em teoria, fornecer informações valiosas (como excluir um *flutter* atrial de 8 dígitos com um istmo comum), mas em nosso centro isso é normalmente realizado apenas se outros métodos não conseguirem explicar o circuito, devido ao pequeno risco de terminar a arritmia ou de sua degeneração em fibrilação atrial.^3^ O mapeamento *Ripple* também pode ser útil nesses casos, pois supera algumas limitações dos mapas de LAT, como a anotação incorreta dos eletrogramas, e o fato de não ser influenciado pela janela de interesse.^4^

Recentemente, um novo algoritmo de mapeamento – *Coherent* (CARTO^®^ 3V7, Biosense Webster) foi desenvolvido para abordar algumas das limitações associadas ao mapeamento de ativação atual. Resumidamente, o algoritmo de mapeamento *Coherent* leva em consideração o valor de LAT, o vetor de condução e a probabilidade de não-condutividade, e exibe o mecanismo de arritmia mais provável.^3^ Ele apresenta alguns novos recursos: 1) A presença dos vetores e sua direção e velocidade correspondentes e 2) Uma zona de “condução lenta ou sem condução” (SNO, *slow or no conduction* ) exibida com uma cor marrom, representando uma área onde há condução lenta ou nenhuma condução.

A análise com este novo algoritmo de mapeamento ( Figura 3B e S-3 B ) permitiu-nos superar algumas das limitações do mapeamento de ativação convencional, mesmo com o recurso de *HD Coloring* : 1) A dificuldade em discriminar entre ativação ativa e passiva – em nosso caso, embora a janela de interesse tenha sido definida como igual à TCL, a parede posterior e o AAE foram ativados com tal atraso que sua ativação continuou no próximo ciclo e, consequentemente, foram exibidos com uma cor vermelha, explicando porque eles pareciam estar simultaneamente ativados; 2) Cada LAT recebe uma anotação única, independentemente de seu fracionamento ou duração, o que pode confundir os operadores e até mesmo o próprio *software* . No presente caso, ao registrar os eletrogramas fracionados na válvula mitral lateral como LAT muito precoces, uma linha branca de bloqueio de condução foi exibida, o que dificultou a interpretação do mapa de ativação. Ao analisar as direções dos vetores e sua velocidade (velocidade lenta se representada por vetores mais grossos), observamos a condução através da parede anterior perto da VPSD – um circuito no sentido horário (seta amarela tracejada na Figura 3B ) ao redor das veias pulmonares direitas. Entretanto, o mapeamento *Coherent* também revelou uma pequena área de condução lenta perto da válvula mitral, sugestiva de um segundo circuito – um *flutter* mitral no sentido anti-horário. Este circuito correspondia à área dos eletrogramas fracionados e poderia ter passado despercebido como uma linha branca, sugerindo que um bloqueio teria sido colocado pelo *HD Coloring* ( Figura 3A ) devido ao motivo mencionado acima (cada LAT recebe um único registro, independentemente de seu fracionamento ou duração) ( Figura 3B , S-3 B e Vídeo Suplementar 3 ).

**Figura 4:**
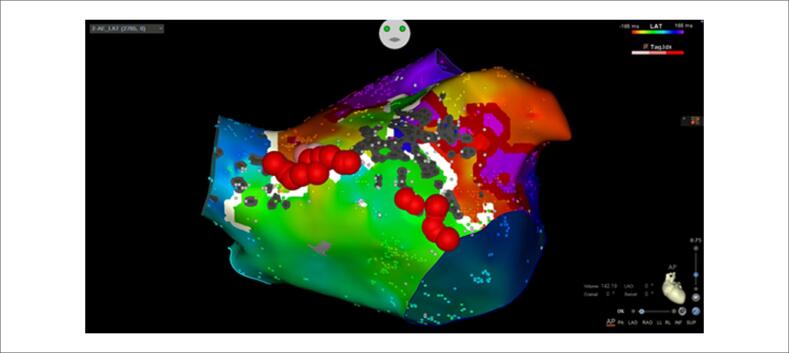
Localização dos sítios de aplicação da radiofrequência, correspondendo aos locais de condução lenta, que permitiram a cessação do flutter atrial com duplo loop.

Para confirmar nossa hipótese de um FLA com *duplo loop* , a etapa final foi a escolha do local para aplicação da energia de radiofrequência (RF). Inicialmente fechamos o gap perto da VPSD com alongamento imediato da TCL ( Figura 1C ). Após a entrega da energia de RF no aspecto anterior da válvula mitral, o FLA foi encerrado com sucesso ( Figura 1D e Figura 4 ). O bloqueio bidirecional ao longo da linha de ablação foi confirmado com manobras de estimulação diferencial e com mapeamento de ativação repetido durante a estimulação do AAE ( Vídeo Suplementar 4 ). Além disso, nenhuma arritmia adicional foi induzida subsequentemente. Após 5 meses de seguimento, o paciente permanece livre de qualquer arritmia sustentada.

Este caso destaca alguns dos novos recursos do algoritmo de mapeamento *Coherent* e sua utilidade, particularmente em pacientes com áreas de cicatriz extensa, eletrogramas fracionados e áreas de condução muito lenta. Ao exibir a de ativação mais lógica, o mapeamento *Coherent* superou as limitações relacionadas com a presença de condução muito lenta e o registro incorreto de eletrogramas fracionados, com a consequente exibição incorreta do bloqueio de condução, revelando um *flutter* atrial com duplo *loop* , e permitindo-nos tratar o paciente com sucesso.

## *Material suplementar

Para verificar as figuras, por favor, clique aqui.



Para assistir ao vídeo suplementar 1, por favor, clique aqui.



Para assistir ao vídeo suplementar 2, por favor, clique aqui.



Para assistir ao vídeo suplementar 3, por favor, clique aqui.



Para assistir ao vídeo suplementar 4, por favor, clique aqui.


