# Supplementary material for: Mapeamento com Software Coherent para Ablação de Flutter Atrial Atípico – Um Passo à Frente na Compreensão do Mecanismo da Arritmia
Source: Arq Bras Cardiol. 2021 Nov 22;117(6):1212–6. [Article in Portuguese] doi: 10.36660/abc.20201311 (PMC8757155; doi:10.36660/abc.20201311)
Supplement: Supplementary file 1 [file 2020-1311-supplemental-material.pdf]

SUPPLEMENTAL MATERIAL

SUPPLEMENTAL FIGURE

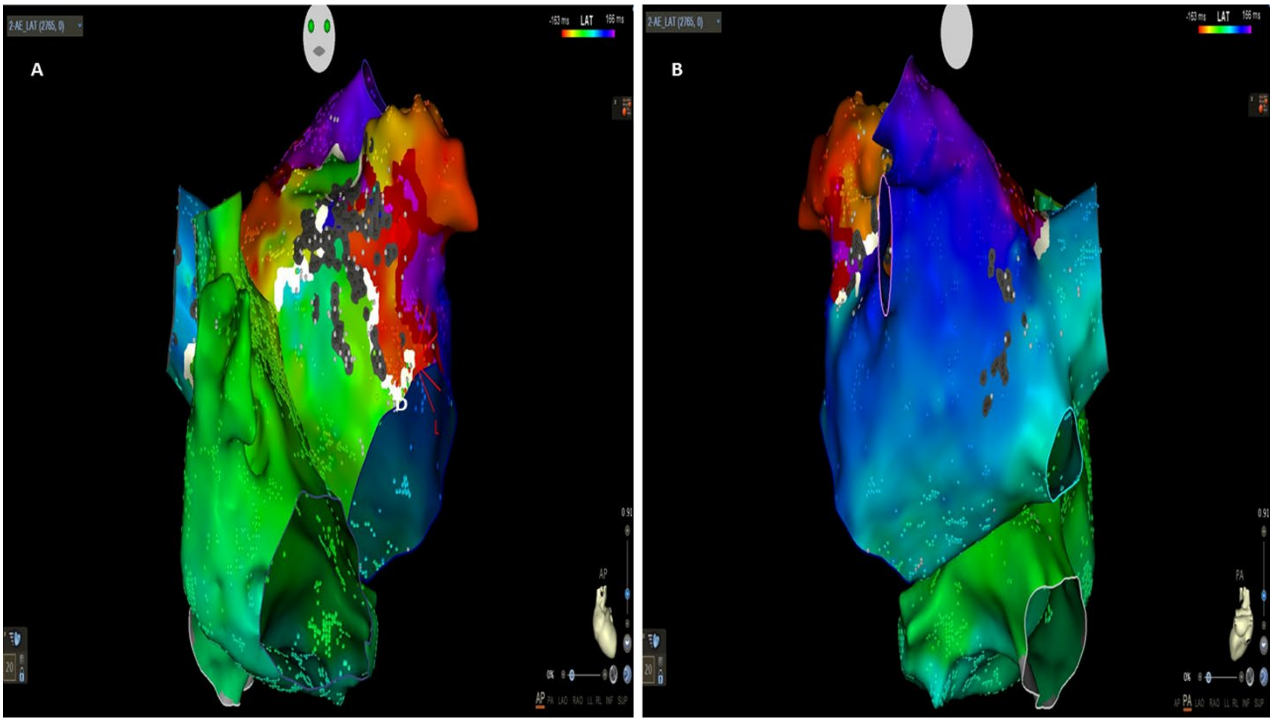

Figure S-1 – Combined high-density activations maps of the RA and LA (A and B) acquired during AFL with a TCL of 330ms and including 7408 points (4643 points in the RA and 2765 points in the LA). Both maps were performed with the HD Coloring software (EML and EEML set at 75% and 25%, respectively), with bipolar scar settings at 0.03mV and scar area size of 1, displayed as grey tags (in the anterior wall and in a small region in the posterior wall near the right inferior pulmonary vein). Red indicates the areas with earliest LAT, while orange, yellow, green, blue and purple indicate progressively delayed activation. The white lines corresponded to lines of probable conduction block, as displayed by an EEML of 25% and the red lines corresponded to areas of reentry (EML ≥ 75%). The combined LAT maps revealed a passive conduction through the RA, several LA areas with early activation points (on the LAA, on the anterior aspect of the MV and on the roof near the anterior segment of the RSPV) and two areas of reentry, displayed at red line – from the LAA to the MV and on the roof. It also revealed a possible line of conduction block (displayed at white) from the MV until almost the RSPV. RA- right atrium; LA- left atrium; AFL –atrial flutter; TCL- tachycardia cycle length;

EML- early meets late; EEML – extended early meets late; LAT – local activation time; LAA – left atrial appendage; MV- mitral valve; RSPV – right superior pulmonary vein.

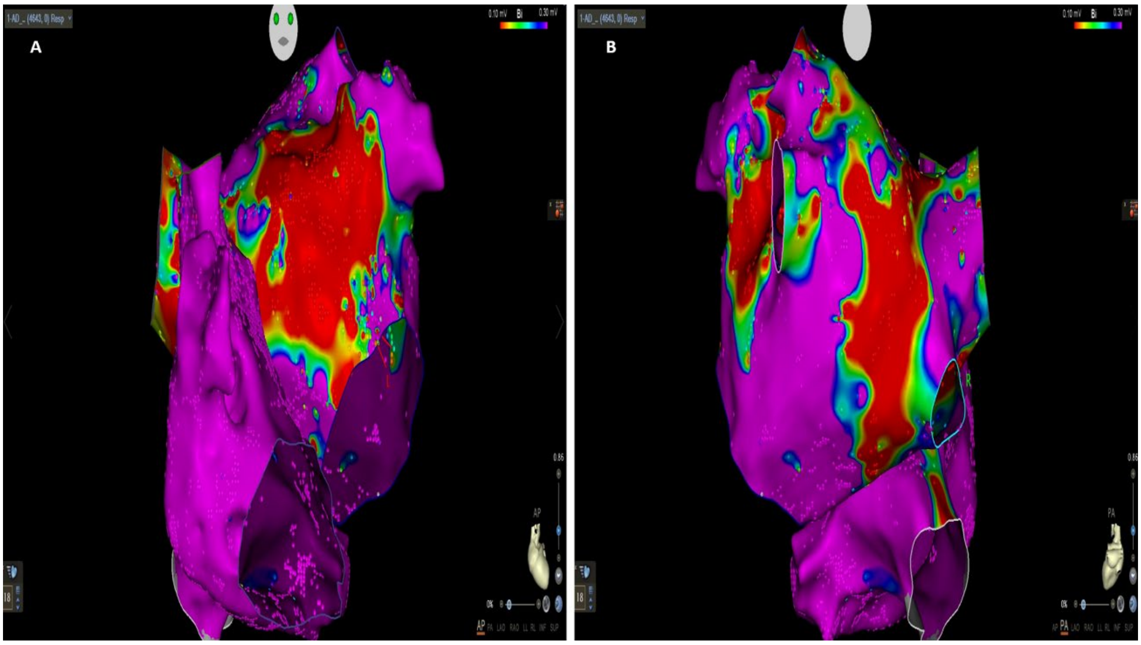

Figure S-2 – High-density bipolar maps of the RA and LA (A, B) acquired during AFL with a TCL of 330ms. All maps were performed with the HD Coloring software. **Maps A and B** represent the bipolar map of both atria and included 7408 points (4643 points in the RA and 2765 points in the LA). In the voltage maps, red indicates areas of probable scar defined as electrogram voltage below 0.1 mV, and purple indicates areas of voltage above 0.3mV. In between, yellow, green and blue represent transition areas. AFL –atrial flutter; LA- left atrium; RA- right atrium; TCL- tachycardia cycle length.

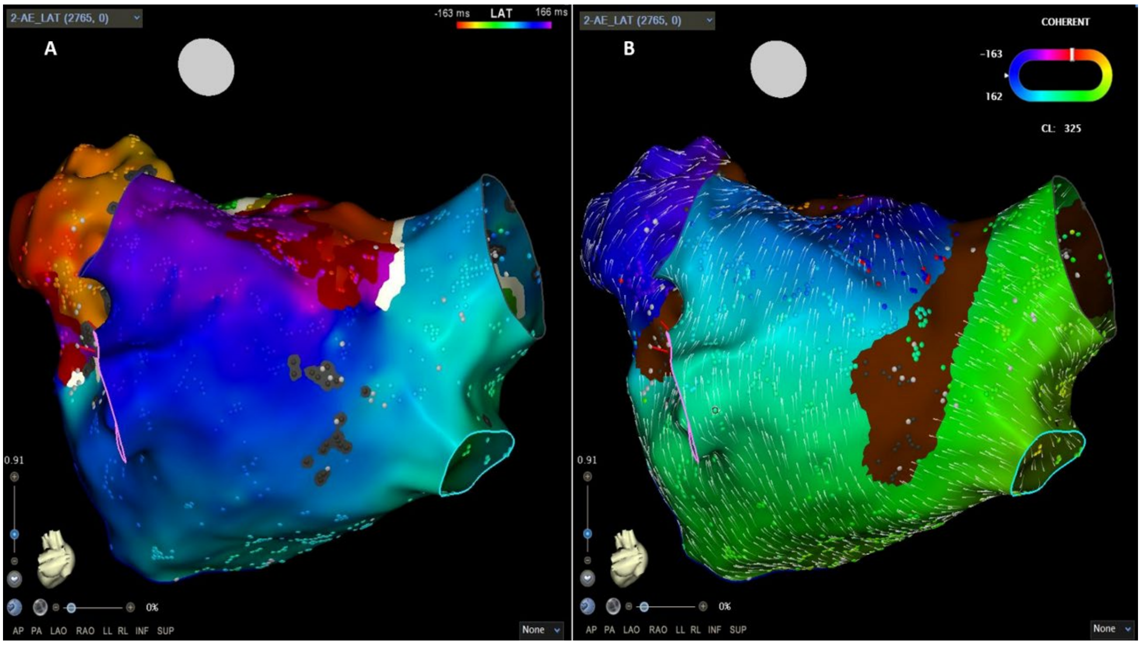

Figure S-3 – Left atrial activation maps (posterior view) performed with the PentaRay®catheter including 2765 points and 330ms of the tachycardia cycle length. **Map A** was performed with the HD Coloring software (EML and EEML set at 75% and 25%, respectively), with bipolar scar settings at 0.03mV and a scar area size of 1 displayed as grey tags. **Map B** was performed with the Coherent mapping algorithm, revealing a SNO (slow or no conduction) zone (displayed as brown) in the posterior wall. EML- early meets late; EEML – extended early meets late.
