# Supplementary material for: Mapeamento com Software Coherent para Ablação de Flutter Atrial Atípico – Um Passo à Frente na Compreensão do Mecanismo da Arritmia
Source: Arq Bras Cardiol. 2021 Nov 22;117(6):1212–6. [Article in Portuguese] doi: 10.36660/abc.20201311 (PMC8757155; doi:10.36660/abc.20201311)
Supplement: Translation

# Translation: Coherent Map for Atypical Atrial Flutter – A Step Forward for the Understanding of the Arrhythmia Mechanism

**Authors:** Pedro A. Sousa, Sérgio Barra, Mariana Pereira, Luís Elvas

**Affiliations:** 1 Coimbra Portugal Pacing & Electrophysiology Unit, Cardiology Department, Centro Hospitalar e Universitário de Coimbra, Coimbra, Portugal, Coimbra – Portugal; 2 Vila Nova de Gaia Portugal Hospital da Luz Arrábida V - Cardiology Department, Vila Nova de Gaia – Portugal; 3 Cambridge United Kingdom Royal Papworth Hospital NHS Foundation Trust – Cardiology Department, Cambridge - United Kingdom

**Keywords:** Atrial Flutter/etiology, Conduction, Arrhytmias, Cardiac, HC Coloring, Coherent Mapping, Electrophysiologic Techniques Cardiac/methods

## Case Presentation

An 86-year-old woman with a previous history of paroxysmal atrial fibrillation was referred for atypical atrial flutter (AFL) ablation. She had never been submitted to catheter ablation before. The transthoracic echocardiogram revealed a left ventricular ejection fraction of 65%, a mildly dilated left atrium (42mm) and moderate mitral regurgitation. The baseline 12-lead ECG exhibited positive F waves in V1 and the lower limb leads, isoelectric F waves in DI and negative F wave in aVL. ( Figure 1A ) Given the earlier concentric activation in the coronary sinus (CS), with a tachycardia cycle length (TCL) of 330ms, ( Figure 1B ) a right atrial (RA) activation map, performed with the PentaRay^®^catheter, was initially analyzed using the HD Coloring tool (CARTO^®^3V7, Biosense Webster, CA, USA). It revealed an earlier activation in the interatrial septum with only 1/3 of the TCL ( Figure 2 ). A subsequent left atrial (LA) activation map revealed a large area of scar with likely conduction block in almost all of the anterior wall, represented as a white line by the lower threshold Extended Early Meets Late (EEML) feature, and several areas with early local activation time (LAT) points – in the left atrial appendage (LAA), the anterior aspect of the mitral valve (MV) and the roof near the anterior segment of the right superior pulmonary vein (RSPV). It also showed two zones of re-entry as established by the Early Meets Late (EML) feature ( Figure 3A and S-3 A ). These maps suggested a probable circuit around the right pulmonary veins but did not explain the color activation in the LAA and MV. A new mapping algorithm – Coherent (CARTO^®^3V7, Biosense Webster) – unveiled the circuit ( Figure 3B and S-3 B ). What is the mechanism of this atypical AFL?

**Figure 1:** Twelve-lead electrocardiogram (A) and intracardiac tracings obtained during AFL (B) and during radiofrequency applications (C and D).

**Figure 2:** High-density activation map of the RA acquired during AFL with a TCL of 330ms. The map was performed with the HD Coloring software, included 4,643 points, with 32% of the TCL and displayed a centrifugal high septal activation (red indicates the areas with earliest LAT, while orange, yellow, green, blue and purple indicate progressively delayed activation). Unipolar electrograms showed an initial “r” deflection. AFL: atrial flutter; RA: right atrium; TCL: tachycardia cycle length.

**Figure 3:** Left atrial activation maps performed with the PentaRay^®^catheter including 2,765 points and 330ms of the tachycardia cycle length. Map A was performed with the HD Coloring software (EML and EEML set at 75% and 25%, respectively), with bipolar scar settings at 0.03mV and a scar area size of 1 displayed as grey tags. The activation map revealed three distinct areas of early LAT points – in the roof, near the anterior MV and in the LAA – and two zones of probable re-entry as defined by a difference between adjacent points superior to 75% of the TCL (the EML threshold) - in the roof and between the LAA and MV, respectively. There was a probable line of conduction block (displayed as a white line) from the MV until almost the RSPV, since the difference between adjacent LAT points on both sides of the line were greater than 25% of the mapped tachycardia cycle. Map B was performed with the Coherent mapping algorithm, revealing a SNO (slow or no conduction) zone (displayed as brown) from the RSPV to the anterior aspect of the mitral valve and also towards the LAA. With the velocity vector set at 17 (slow velocity is represented by thicker vectors), we observed conduction through the anterior wall near the RSPV - a clockwise circuit (dashed yellow arrow) around the right pulmonary veins. Coherent also revealed a small area of slow conduction (fractionated electrograms as depicted in Map A) near the mitral valve suggestive of a second circuit - a counterclockwise propagation wave around the MV (dashed yellow arrow). EML: early meets late; EEML: extended early meets late; LAT: local activation time; MV: mitral valve; LAA: left atrial appendage; TCL: tachycardia cycle length; RSPV: right superior pulmonary vein.

## Discussion

This case highlights some interesting features.

First, taking into consideration the concentric activation in the CS ( Figure 1B ), the mapping was initially performed in the RA with the HD Coloring software. The bipolar map revealed normal electrograms, defined as voltage above 0.3mV, in most of the chamber ( Figure S-2 A and S-2 B ). A RA high-density activation map revealed earliest LAT points in the interatrial septum ( Figure 2 ). The propagation wave at this point was consistent with a focal source, but since the unipolar signal had an initial “r” deflection and only 1/3 of the TCL was comprised in this chamber, an exclusively RA circuit was ruled out ( Supplemental Video 1 ).

An LA bipolar map was subsequently performed, revealing a diseased LA, with extensive dense scarring in the anterior wall, as defined by a voltage below 0.1mV, and some patchy scar areas in the posterior wall ( Figure S-2 A and S-2 B ). The high-density activation map performed with the HD Coloring software ( Figure 3A and S-3 A ), comprising all of the TCL, displayed multiple areas of early activation (in the LAA, the anterior aspect of the MV and the roof near the anterior segment of the RSPV). Two areas of re-entry, as defined by the EML feature, were also observed – one from the LAA to the MV and another in the roof. Both the EML and EEML depend on the mapped tachycardia cycle (which in our case corresponded to the TCL) and they are established taking into consideration the LAT difference between adjacent points.^1 , 2^In this case, since the difference between LAT points was greater than 25% of the TCL (82ms = 0.25 * 330ms), a white line was displayed from the MV to the RSPV (sparing only a small portion in the anterior wall, near the RSPV) and also in the posterior wall near the RSPV, suggesting probable lines of conduction block.

The propagation map of the LA ( Supplemental Video 2 ) suggested a circuit around the right pulmonary veins, with the propagation wave moving through the interrupted white line. However, it did not explain the color activation in the LAA and MV. Even after combining both maps ( Figure S-1 ), several questions remained unexplained:

1. How could there be a propagation wave in the anterior wall given the presence of extensive scar?
2. How was there a simultaneous activation of different areas of the LA?
3. Do the two areas of reentry – in the posterior roof and from the LAA to the MV- correspond to two independent circuits or one circuit with passive conduction to the other area?
4. In case of independent circuits, where are they located and how can the wavefront propagate through the anterior aspect of the MV if, according to the EEML, this appears blocked (as shown by the uninterrupted white line)?

As previously reported,^2^in the HD Coloring feature each LAT requires a voltage of only ≥ 0.03mV to be integrated into the activation map. This is the reason why we could see an activation wavefront in the anterior wall, despite the presence of extensive scar (bipolar electrogram voltage below 0.1mV). Entrainment could in theory provide valuable information (like excluding an 8-digit atrial flutter with a common isthmus), but in our center this is typically performed only if other methods fail to explain the circuit, given the small risk of terminating the arrhythmia or its degeneration into atrial fibrillation.^3^Ripple mapping can also be useful in these cases, since it overcomes some limitations of the LAT maps, such as the incorrect annotation of the electrograms and the fact that it is not influenced by the window of interest.^4^

Recently, a novel mapping algorithm – Coherent (CARTO^®^3V7, Biosense Webster) was developed to address some of the limitations associated with the current activation mapping. Briefly, the Coherent mapping algorithm takes into account the LAT value, the conduction vector and the probability of non-conductivity and displays the most likely arrhythmia mechanism.^3^It introduces some new features: 1) The presence of the vectors and their corresponding direction and velocity and 2) A “slow or no conduction” (SNO) zone displayed with a brown color, representing an area where there is slow conduction or no conduction.

The analysis with this new mapping algorithm ( Figure 3B and S-3 B ) allowed us to overcome some of the limitations of conventional activation mapping, even with the HD coloring feature: 1) The difficulty in discriminating between active and passive activation – in our case, although the window of interest was defined as equal to the TCL, the posterior wall and the LAA were activated with such delay that its activation continued into the next cycle and consequently were displayed with a red color, explaining why they seemed to be simultaneously activated; 2) Each LAT receives one single time annotation regardless of its fractionation or duration, which can mislead the operators and even the software itself. In the present case, by annotating the fractionated electrograms in the lateral mitral valve as very early LAT, a white line of conduction block was displayed, which hindered the interpretation of the wavefront activation map. By analyzing the vectors’ directions and their velocity (slow velocity if represented by thicker vectors) we observed conduction through the anwterior wall near the RSPV – a clockwise circuit (dashed yellow arrow in Figure 3B ) around the right pulmonary veins. However, Coherent mapping also revealed a small area of slow conduction near the mitral valve, suggestive of a second circuit – a counterclockwise mitral flutter. This circuit corresponded to the area of fractionated electrograms and could have gone unnoticed as a white line, suggesting that a block had been placed by HD Coloring ( Figure 3A ) for the reason mentioned above (each LAT receives one single time annotation regardless of its fractionation or duration) ( Figure 3B , S-3 B and Supplemental Video 3 ).

To confirm our hypothesis of a double loop AFL, the final step was the choice of location for the application of radiofrequency (RF) energy. We initially closed the gap near the RSPV with immediate elongation of the TCL ( Figure 1C ). After delivery of RF energy in the anterior aspect of the mitral valve, the AFL was successfully terminated ( Figure 1D and Figure 4 ). Bidirectional block along the ablation line was confirmed with differential pacing maneuvers and with repeat activation mapping while pacing from the LAA ( Supplemental Video 4 ). Also, no further arrhythmia was subsequently inducible. After 5 months of follow-up, the patient remains free from any sustained arrhythmia.

**Figure 4:** Location of the sites of radiofrequency delivery, corresponding to the places of slow conduction, which allowed the double loop atrial flutter termination.

This case highlights some of the new features of the Coherent mapping algorithm and its usefulness, particularly in patients with extensive scar, fractionated electrograms and areas of very slow conduction. By displaying the “best fit” activation wavefront, Coherent mapping overcame the limitations related with the presence of very slow conduction and the incorrect annotation of fractionated electrograms with incorrect consequent display of conduction block, unveiling a double loop atrial flutter, and allowing us to successfully treat the patient.

## *Supplemental Materials

For supplement figures, please click here



See the Supplemental Video 1, please click here.



See the Supplemental Video 2, please click here.



See the Supplemental Video 3, please click here.



See the Supplemental Video 4, please click here.
